# Application of Geographically Weighted Regression Analysis to Assess Predictors of Early Sexual Initiation in Ethiopia, EDHS 2016

**DOI:** 10.1002/hsr2.72177

**Published:** 2026-03-22

**Authors:** Shimels Derso Kebede, Adamu Ambachew Shibabaw

**Affiliations:** ^1^ Department of Health Informatics, School of Public Health, College of Medicine and Health Science Wollo University Dessie Ethiopia; ^2^ Department of Health Informatics, College of Health Science Mettu University Mettu Ethiopia

**Keywords:** early sexual initiation, EDHS, Ethiopia, geographically weighted regression (GWR)

## Abstract

**Background and Aims:**

Early sexual initiation was defined as the experience of first intercourse before 18 years of age. It has negative health, social, and economic consequences with an increased lifetime prevalence of sexual partners, thereby increasing the risk of exposure to sexually transmitted diseases. Thus, this study aimed to assess the spatial relationship of factors associated with early sexual initiation and identify its predictors in Ethiopia.

**Methods:**

The data were obtained from the Measure demographic and health survey website and extracted through SPSS and Stata software. To account for disproportionate sampling and non‐response, sample weights were applied. A total weighted samples of 15,683 women were selected and the DHS used a stratified, two‐stage cluster sampling technique. Six hundred forty‐five enumeration areas with latitude and longitude coordinates were used. ArcGIS version 10.5 software was used to analyse spatial statistics. The geographically weighted regression analysis technique was used to identify predictors of early sexual initiation.

**Result:**

The result of geographically weighted regression analysis has identified different variable coefficients through different geographical locations. Accordingly, higher coefficients for rural residence were detected in Dire Dawa, Harari region, north‐eastern part of Oromia and northern part of Somali, The other spatial predictor was poorest wealth index and its higher coefficients were detected in Addis Ababa, Benishangul Gumuz, western part of Oromia, northern part of SNNP and Amhara region, and being from female headed household was also an important predictor with higher coefficients detected in Afar, all parts of Tigray region, the northern part of Amhara region, Benishangul Gumuz region and Wellga zone of Oromia.

**Conclusion:**

The study revealed significant spatial variation in predictors of early sexual initiation, with rural residence, poorest wealth index, and female‐headed households showing stronger effects in specific regions. High‐risk areas include Dire Dawa, Harari, parts of Oromia, Addis Ababa, Benishangul Gumuz, and Tigray. Targeted, region‐specific interventions focusing on poverty reduction, rural youth support, and empowerment of female‐headed households are essential to address early sexual initiation effectively.

**Abbreviations**
AIC: akaike information criteria
DHS: demographic and health survey
EDHS: Ethiopian demographic and health survey
GWR: geographically weighted regression
HPV: human papillomavirus
OLS: ordinary least square
SNNPR: Southern Nations, Nationalities and Peoples Region
SPSS: Statistical Package for Social Sciences
VIF: variance inflation factor

## 1 Introduction

Different scholars define early sexual initiation according to the social and demographic context of the nation [1, 2, 3]. But, the Universal Declaration of Human Rights proclaimed as an age below 18 years old is considered a child, they couldn't decide concerning marriage and/or consensual sexual relationship [4]. Early age sexual initiation was defined as the experience of first intercourse before 18 years of age [5].

Early sexual initiation has negative health, social, and economic consequences for both women and future generations. It is associated with an increased lifetime prevalence of sexual partners, thereby increasing the risk of exposure to sexually transmitted diseases, including HIV/AIDS, and pregnancy. The early sexual debut also increases the risk of human papillomavirus (HPV) infection, due to cervical immaturity; and thus the risk of cervical cancer increases [6]. Additionally, given the risk of pregnancy, early sexual initiators are less likely to complete their schooling thereby limiting their social and vocational futures. Youth who begin early sexual activity are more likely to practice risky sexual behaviors, such as multiple sexual partners and incorrect or inconsistent condom use. As a result, they increase the risk of unsafe abortion, early childbirth, and psychosocial problems. These problems are the greatest threats to the health and well‐being of the youth [7, 8]. It also increased the risk of school dropout, poor school performance, stigma, and discrimination [9, 10, 11]. Furthermore, it affects the social and economic status during adulthood [12].

Various researches have been done on the prevalence and factors associated with early sexual initiation in Ethiopia. Age, residence, educational status, parent‐youth discussion, using addictive substances and religion were determinant factors identified [13, 14, 15, 16, 17, 18, 19]. Furthermore, the prevalence and spatial distribution of early sexual initiation were assessed, and hotspot areas were identified [5]. However, to identify predictors of early sexual initiation all the studies including [5] were analyzed with standard logistic regression which is incapable of addressing the flexible relationship of variables across different locations in the study area. Understanding and identifying factors associated with early sexual initiation in specific location is essential for developing effective policies and strategies to reduce the adverse consequences specific to strong factor for that specific area. Therefore, this study aimed to assess the spatial relationship of factors associated with early sexual initiation and identify variables that are strong predictors of early sexual initiation in different parts of the study area through the application of geographically weighted regression (GWR) analysis method. The findings of this study will help policymakers, program planners, non‐governmental organizations, and other health care programs in guiding plan and prioritize prevention and intervention programs based on identified predictors in different hotspots of early sexual initiation.

## 2 Methodology

### 2.1 Study Setting

**Figure 1:**
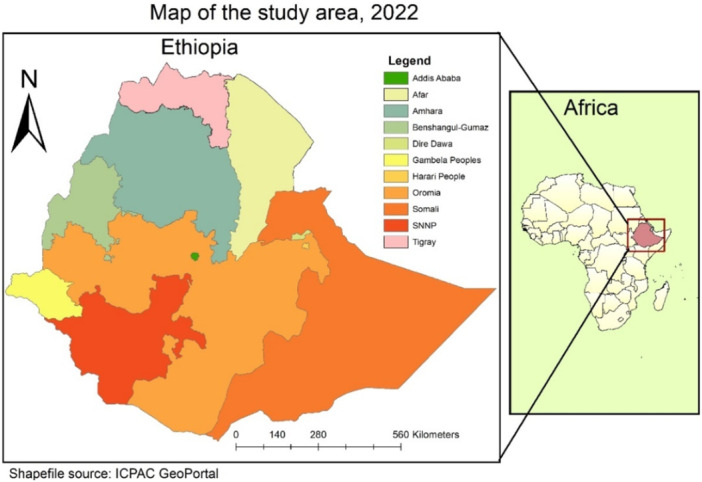
Map of the study area.

The study was conducted in Ethiopia, which is located in North‐eastern Africa which lies between 30 and 150 North latitude and 330 480 and East longitudes. Ethiopia, officially the Federal Democratic Republic of Ethiopia, is a landlocked country in the Horn of Africa (Figure 1). It shares borders with Eritrea and Djibouti to the north, Somalia to the east and northeast, Kenya to the south, South Sudan to the west, and Sudan to the northwest. Ethiopia has a total area of 1,100,000 square kilometers (420,000 sq mi). It has 11 administrative regions and 2 City administrations divided into 817 districts and 16,253 kebeles (Figure 1).

### 2.2 Data and Population

The data were obtained from the 2016 Ethiopian demographic and health survey (EDHS) dataset and data extraction were done through Statistical Package for Social Sciences (SPSS) software. The extracted data were exported into STATA (.dta) file and data weighting, data cleaning, and recoding were performed using STATA version 16.0 and Microsoft Office Excel 2016 Software. The sample distribution to different regions, as well as urban and rural areas, was not proportional in the Ethiopian demographic and health survey dataset. To account for disproportionate sampling and non‐response, sample weights were applied.

A total weighted sample of 15,683 women was considered for the entire analysis. Since this study is based on secondary data from the 2016 Ethiopian Demographic and Health Survey, all cases of reproductive age women aged 15–49 who had complete information on age at first sexual intercourse were included in the analysis. To select study participants, EDHS used a stratified and two‐stage cluster sampling technique was employed. In the first stage, enumeration areas (EAs) were selected from the national census sampling frame using probability proportional to size. In the second stage, a fixed number of households were systematically selected from each EA.

Six hundred forty‐five enumeration areas with latitude and longitude coordinates were used for the 2016 EDHS. Geographical location data (latitude and longitude coordinates) were also taken from selected enumeration areas. Of those enumeration areas, 23 enumeration areas had no latitude and longitude coordinates. After removing those missed location data enumeration areas total of 622 enumeration areas were further used for analysis. Location data values were shifted 1–2 km for urban and 5 km for rural areas for data confidentiality reasons.

### 2.3 Study Variables

EDHS variable age at first sex, a continuous variable, was categorized into two categories: age above and below 18 years. The outcome variable was early sexual initiation represented as a continuous variable of the frequency of women who were engaged in sexual intercourse before the age of 18. Otherwise, women who were engaged in sexual intercourse after the age of 18 were labeled as late sexual initiation. Socio‐demographic and sexual characteristics variables such as: region, religion, residence, wealth index, women's education, sex of household head, working status, marital status, pregnancy, abortion, respondent's circumcision, smoking, frequency of reading newspapers or magazines, use of the internet, and ownership of a mobile telephone were independent variables.

### 2.4 Spatial Analysis

ESRI's ArcGIS version 10.5 software was used to visualize maps and analyse spatial statistics. Ordinary least squares (OLS) and GWR regression analysis techniques were used for identifying factors associated with early sexual initiation.

### 2.5 OLS Regression

Spatial regression modeling was performed to identify predictors of early sexual initiation. Before conducting the final OLS regression, we employed Exploratory Regression analysis using ArcGIS software to systematically assess and identify the most suitable set of explanatory variables. This data mining technique evaluates all possible combinations of candidate independent variables to determine which models meet the essential OLS assumptions, including statistical significance of coefficients (*p* < 0.05), absence of multicollinearity (variance inflation factor [VIF] < 7.5), normal distribution of residuals (Jarque‐Bera, *p* > 0.05), and spatial randomness of residuals (Global Moran's I, *p* > 0.05). Variables that failed to satisfy any of these diagnostic criteria were excluded from the final OLS model. This rigorous pre‐selection process ensured that the final regression results were both statistically sound and theoretically justifiable.

Following this, the final OLS regression was conducted first OLS regression was conducted using the subset of variables identified through exploratory regression, allowing for a more robust and interpretable model of the factors associated with early sexual initiation. Findings from the OLS regression are only reliable if the regression model satisfies all of the assumptions that are required by this method. The coefficients of explanatory variables in a properly specified OLS model should be statistically significant and have either a positive or negative sign. Besides, there should not be a correlation among explanatory variables (free from multicollinearity). The model should be unbiased (heteroscedasticity or non‐stationarity). The residuals should be normally distributed and revealed no spatial patterns. The model should include key explanatory variables. The residuals must be free from spatial autocorrelation. Thus, these assumptions were checked accordingly.

To identify a model that fulfils the assumption of the OLS method, exploratory regression identifies models with high Adjusted *R* ^2^ values, lower akaike information criteria (AIC) and less than 7.5 VIF values. Besides, it identifies models that meet all of the assumptions of the OLS method.

### 2.6 GWR

A variable that is a strong predictor in one cluster may not necessarily be a strong predictor in another cluster. This type of cluster variation (non‐stationary) can be identified through the use of GWR. In this context, GWR can help to answer the question: “Does the association vary across space?” Unlike OLS which fits a single linear regression equation to all of the data in the study area, GWR creates an equation for each DHS cluster. While the equation in OLS is calibrated using data from all features (cluster in this case), GWR uses data from nearby features. Thus, the GWR coefficient takes different values for each cluster Maps of the coefficients associated with each explanatory variable, which are produced using the GWR, provide guidelines for targeted interventions.

### Ethics Approval and Consent to Participate

Permission to use the data has been granted by the Measure DHS program through legal registration. EDHS (2016) data were used which is available on the public domain through the Measure DHS website (www.measuredhs.com). Consent for publication is not applicable, as the study utilized secondary data from the Ethiopian Demographic and Health Survey, and no data were collected directly from individuals by the authors.

## 3 Results

### 3.1 Sociodemographic and Socioeconomic Characteristics

Among a total of 15,683 respondents, 41% of women were Orthodox (6413) followed by 39.5% Muslim respondents (6209). Out of all respondents, 10,335 (66%) were rural residents and nearly half (45%) of the respondents were without any formal education. Most of the respondents, 10,011 (64%) were non‐working women and about were married (63%). More than half, 9841 (63%) of respondents have no mobile phone and almost all 14,328 (91%) don't use the internet in their daily life. Regarding substance use, almost all (99%) of the respondents were not smokers (Table 1).

**Table 1:** Socio‐demographic characteristics of reproductive‐age women respondents, EDHS 2016 (*n* = 15,683).

Variable	Sexual initiation		
Religion	Early sexual initiation	Late sexual initiation	Total
Orthodox	3494 (54.50%)	2919 (45.50%)	6413
Catholic	48 (52.75%)	43 (47.25%)	91
Protestant	1514 (53.79%)	1300 (46.21%)	2814
Muslin	3840 (61.86%)	2369 (38.14%)	6209
Traditional	57 (67.86%)	27 (32.14%)	84
Others	44 (61.11%)	28 (38.89%)	72
Region			
Tigray	1030 (61.26%)	652 (38.74%)	1682
Afar	839 (74.38%)	289 (25.62%)	1128
Amhara	1211 (70.40%)	508 (29.60%)	1719
Oromia	1167 (61.69%)	725 (38.31%)	1892
Somali	756 (54.37%)	635 (45.63%)	1391
Benishangul Gumuz	738 (65.56%)	388 (34.44%)	1126
SNNPR	927 (50.14%)	922 (49.86%)	1849
Gambela	703 (67.88%)	332 (32.12%)	1035
Harari	505 (55.74%)	401 (44.26%)	906
Addis Ababa	563 (30.88%)	1261 (69.12%)	1824
Dire Dawa	558 (49.34%)	573 (50.66%)	1131
Residence			
Urban	2225 (41.61%)	3123 (58.39%)	5348
Rural	6772 (65.53%)	3563 (34.47%)	10,335
Wealth index			
Poorest	2677 (68.73%)	1217 (31.27%)	3894
Poorer	1400 (68.42%)	646 (31.58%)	2046
Middle	1268 (63.32%)	734 (36.68%)	2002
Richer	1210 (59.23%)	832 (40.77%)	2042
Richest	2442 (42.85%)	3257 (57.15%)	5699
Educational level			
No education	5342 (75.97%)	1691 (24.03%)	7033
Primary education	2647 (50.77%)	2566 (49.23%)	5213
Secondary and above	1008 (29.34%)	2429 (70.66%)	3437
Working status			
Not working	5828 (58.19%)	4183 (41.81%)	10,011
Working	3169 (55.86%)	2503 (44.14%)	5672
Marital status			
Never married	277 (6.48%)	4001 (93.52%)	4278
Married	7488 (76.22%)	2336 (23.78%)	9824
Widowed	1232 (77.92%)	349 (22.08%)	1581
Read newspaper/magazine			
No	8163 (62.28%)	4943 (37.72%)	13,106
Yes	834 (32.36%)	1743 (67.64%)	2577
Use Internet			
No	8726 (60.87%)	5603 (39.13%)	14,329
Yes	271 (20.01%)	1083 (79.99%)	1354
Own mobile phone			
No	6397 (65.00%)	3444 (35.00%)	9841
Yes	2600 (44.50%)	3242 (55.50%)	5842
Smoker			
No	8903 (57.23%)	6650 (42.77%)	15,553
Yes	94 (72.31%)	36 (27.69%)	130
Sex of household head			
Male	6503 (59.93%)	4350 (40.07%)	10,853
Female	2494 (51.65%)	2336 (48.35%)	4830

### 3.2 Sexual Related Characteristics

About 14,561 (93%) of the respondents were not pregnant during the survey and 14,447 (92%) had not any previous abortion history. Furthermore, about 4984 (32%) respondents were circumcised and 5409 (34%) were women with no children (Table 2).

**Table 2:** Sexual characteristics of reproductive‐age women respondents, EDHS 2016 (*n* = 15,683).

Variable	Sexual initiation		
Pregnancy status	Early sexual initiation	Late sexual initiation	Total
Currently not pregnant	8160 (56.05%)	6401 (43.95%)	14,561
Currently pregnant	837 (74.58%)	285 (25.42%)	1122
Ever aborted			
No	8032 (55.60%)	6415 (44.40%)	14,447
Yes	965 (78.09%)	271 (21.91%)	1236
Respondent circumcised			
Not circumcised	972 (44.61%)	1207 (55.39%)	2179
Circumcised	3107 (62.36%)	1877 (37.64%)	4984
Children ever born			
No children	978 (18.08%)	4431 (81.92%)	5409
1–3 children	3658 (71.45%)	1462 (28.55%)	5120
4–6 children	2663 (83.08%)	542 (16.92%)	3205
4 and more children	1698 (87.09%)	251 (12.91%)	1949

### 3.3 OLS Regression Analysis

During the first exploratory regression independent variables like currently not pregnant, not working, never used internet, not read news, not owning mobile phone, no education and rural residence were found to be correlated (VIF > 7.5) which means all are telling the same story about early sexual initiation.

After removing those multi‐correlated variables, when the second exploratory regression runs a model with seven independent variables such as rural residence, poorest wealth index, female HH, never married, circumcised and smoking was found to be a candidate model for OLS regression with an adjusted *R* ^2^ of 0.85, AIC of 4230 and 2.84 VIF value.

Based on the results from exploratory regression, OLS regression was run, and the six requirements of OLS were checked (Table 3).

**Table 3:** Summary of OLS results for early sexual initiation in Ethiopia, EDHS 2016.

Variable	Coefficient	Standard error	*t*‐Statistic	Probability	Robust standard error	Robust *t*‐statistic	Robust probability	VIF
Intercept	0.51	0.42	1.21	0.23	0.36	1.43	0.15	….
Rural residence	0.43	0.02	27.14	0.00*	0.04	12.15	0.00*	2.35
Poorest wealth index	0.22	0.04	5.58	0.00*	0.07	3.24	0.001*	1.44
Female household head	0.69	0.06	12.02	0.00*	0.10	6.62	0.00*	2.60
Never married	−0.13	0.06	−2.29	0.02*	0.12	−1.03	0.30	2.83
circumcised	0.01	0.02	0.17	0.86	0.03	0.16	0.87	1.00
Being smoker	0.64	0.29	2.23	0.03*	0.62	1.04	0.29	1.05
OLS diagnostics								
Number of observations:	622	Akaike's information criterion (AICc)				4230.95		
Multiple *R*‐squared [d]:	0.86	Adjusted *R*‐squared [d]:				0.85		
Joint *F*‐statistic [e]:	517.56	Prob(> *F*), (7614) degrees of freedom:				0.000000*		
Joint Wald statistic [e]:	1173.88	Prob(> chi‐squared), (7) degrees of freedom:				0.000000*		
Koenker (BP) statistic [f]:	140.69	Prob(> chi‐squared), (7) degrees of freedom:				0.000000*		
Jarque‐Bera statistic [g]:	2187.52	Prob(> chi‐squared), (2) degrees of freedom:				0.000000*		

Abbreviations: EDHS, Ethiopia Demographic and Health Survey; VIF, variance inflation factor.

* Significant variables under 95% confidence interval.

#### 3.3.1 Check 1: Model Performance

The model yields 0.853 adjusted *R* ^2^ and 4230.95 AIC. This result is interpreted as the model explained about 85% of the variance in early sexual initiation.

#### 3.3.2 Check 2: Model Significance

Joint Wald statistic was used to check the model significance. Results showed that the overall model is statistically significant with *p*‐value < 0.05.

#### 3.3.3 Check 3: Coefficients of Exploratory Variables

Coefficient, Robust Probability, and VIF of each exploratory variable were assessed. Rural residence, poorest wealth index, and female household head were factors significantly associated with early sexual initiation with robust probability of < 0.05. All exploratory variables have VIF value less than 7.5. All significant variables have also positive relationship with early sexual initiation. Respondents who are rural resident, had poorest wealth index, living in female headed household could increase early sexual initiation by 0.43, 0.22, and 0.83 times.

#### 3.3.4 Check 4: Model Stationarity

The Koenker (BP) Statistic is a test to determine whether the explanatory variables in the model have a consistent relationship to the dependent variable across space. A statistically significant value of Koenker (BP) Statistic means there is nonstationary. Results from OLS show that the relationship between exploratory variables and early sexual initiation has variation across different enumeration areas. This result also indicated us to use robust probability for exploratory variable significance test and Joint Wald statistic for assessing model significance.

#### 3.3.5 Check 5: Model Bias

This check test whether the residuals are normally distributed or not using the Jarque–Bera statistic. Results of this test shows significant Jarque–Bera statistic (*p* < 0.05) which means the residuals are not normally distributed, indicating your model is biased.

#### 3.3.6 Check 6: Residuals Autocorrelation

**Figure 2:**
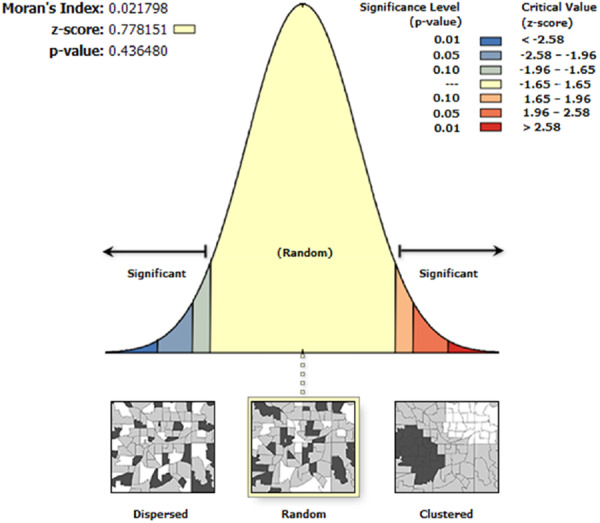
Residual autocorrelation.

Spatial Autocorrelation analysis on the regression residuals ensured that they are spatially random with *Z*‐score 0.78 and *p*‐value of 0.44 (Figure 2).

### 3.4 GWR Analysis

Since the OLS diagnostics indicate significant non‐stationarity, there is a spatially varying relationship among variables, which can be better addressed through GWR analysis. The GWR regression with significant variables from OLS analysis such as rural residence, poorest wealth index, and female household head have produced adjusted *R* ^2^ of 0.89 and 4063.34 AIC value. The adjusted *R* ^2^ value obtained from OLS increased by 4% from 0.85 to 0.89 (Table 4).

**Table 4:** Geographic weighted regression (GWR) model diagnostics for early sexual initiation in Ethiopia, EDHS 2016.

Explanatory variables	Rural residence, poorest wealth index, and female household head
Bandwidth	1.50
Residual squares	21,125.84
Effective number	64.70
Sigma	6.16
Akaike's information criterion (AICc)	4063.34
Multiple *R*‐squared	0.90
Adjusted *R*‐squared	0.89

This was further supported by a corrected Akaike's Information Criterion value where GWR provided a smaller AIC value (4063.34) as compared to a global model (OLS) AIC value which was 4235.85 (Table 5). If the AIC values for two models (OLS and GWR) differ by more than 3, the model with the lower AIC is considered to be better. This indicates that GWR is better than OLS spatial analysis for modeling early sexual initiation.

**Table 5:** Model comparison of OLS and GWR model.

Exploratory variables	OLS result	GWR result
Rural residence Poorest wealth index Female HH	*Adjusted R^2^:* 0.85 *AIC:* 4235.85	*Adjusted R^2^:* 0.89 *AIC:* 4063.34

**Figure 3:**
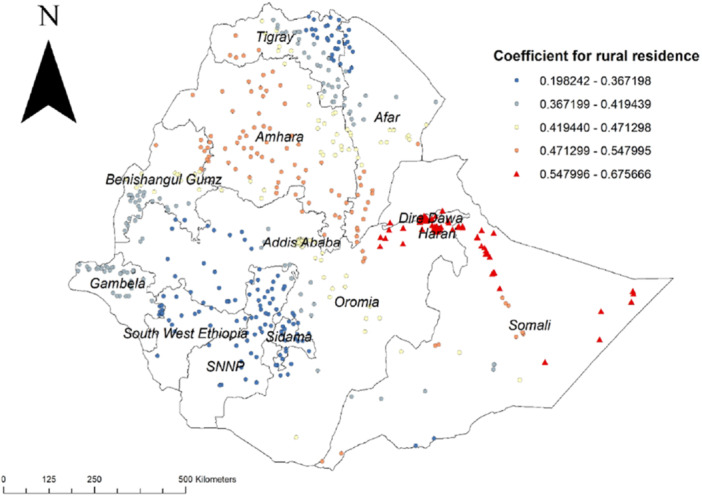
GWR coefficient estimates for rural residence as a predictor of early sexual initiation in Ethiopia, EDHS 2016.

The result of the GWR analysis has identified different variable coefficients across different geographical locations. Accordingly, higher coefficients for rural residence were detected in Dire Dawa, Harari region, the north eastern part of Oromia, and the northern part of Somali (Figure 3).

**Figure 4:**
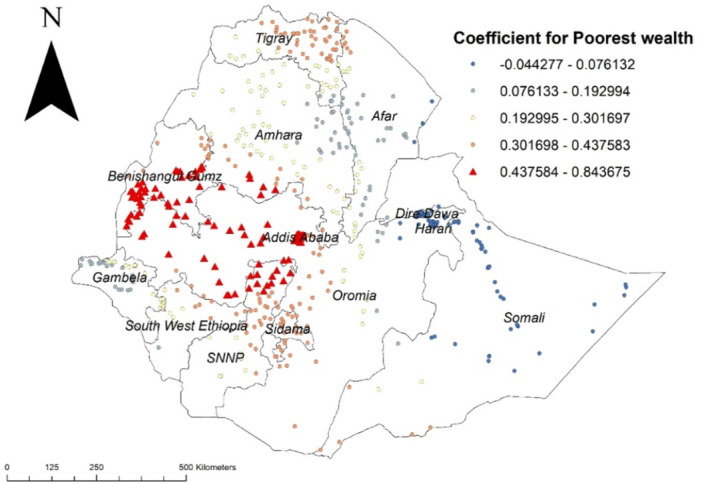
GWR coefficient estimates for poorest wealth index predictor of early sexual initiation in Ethiopia, EDHS 2016.

The other important spatial predictor of early sexual initiation was the poorest wealth index, and its higher coefficients were detected in Addis Ababa, Benishangul Gumz, the western part of Oromia, the northern part of SNNPR, and the north Gojjam zone of the Amhara region (Figure 4).

**Figure 5:**
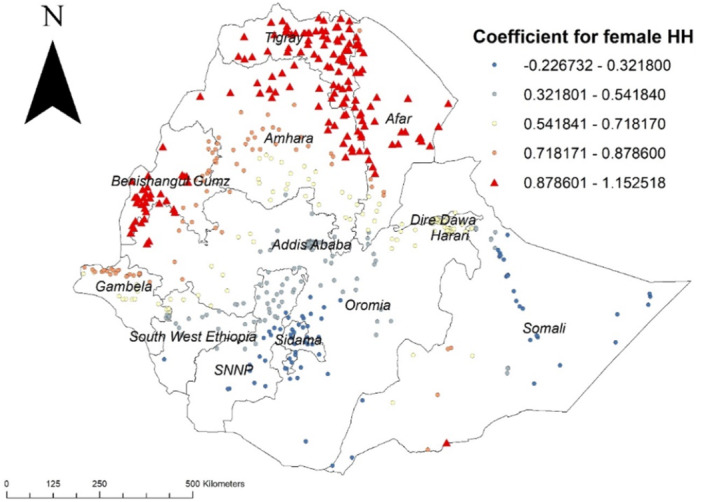
GWR coefficient estimates for women from female household head predictor of early sexual initiation in Ethiopia, EDHS 2016.

Similarly, female household head was also a factor for early sexual initiation and higher coefficients were detected in Afar, all parts of Tigray region, the northern part of Amhara region, Benishangul Gumz region and wellga zone of Oromia region (Figure 5).

## 4 Discussion

This study aimed to assess the spatial relationship of factors associated with early sexual initiation and identify variables that are strong predictors of early sexual initiation in Ethiopia using advanced statistical models. In spatial regression analysis, different statistically significant predictors of early sexual initiation were identified. Accordingly, rural residence, poorest wealth index, and female household head were factors significantly associated with early sexual initiation. The findings of the current study can be used to prioritize the expansion of prevention programs such as health education to the most affected areas of Ethiopia.

Rural residences had a strong relationship with early sexual initiation in Dire Dawa, Harari, and Somali regions. This finding is consistent with previous studies [5, 20, 21, 22]. This could be explained by a lack of community awareness of concerns related to reproductive health and the negative effects of early marriage for rural adolescents [23]. In addition, this discrepancy between urban and rural locations may be caused by urban residents’ better access to healthcare services. Through mass media like television, periodicals, and the internet, people who live in urban areas are more exposed to various sexual and reproductive health‐related information [24]. To address the high rates of early sexual initiation in rural areas, the implementation of comprehensive sexual education programs focused on delaying sexual initiation, promoting reproductive health awareness, and encouraging informed decision‐making among rural youth could be effective.

Similarly, when compared to respondents from the wealthiest wealth index, having a poorer wealth index increases the likelihood of initiating sexual activity earlier in Addis Ababa, Benishangul Gumz, the western part of Oromia, the northern part of SNNPR, and the north Gojjam zone of Amhara region. This result is in line with previous studies [25, 26, 27]: In fact, low socioeconomic women may have early sex in exchange for money and other benefits. In other words, poverty has a negative impact on teenagers’ future because it causes problems with their sexual and reproductive systems.

Being from female headed household was also a factor for early sexual initiation and higher coefficients were detected in Afar, all parts of the Tigray region, the northern part of the Amhara region, the Benishangul Gumz region, and wollega zone of the Oromia region. This finding is in line with previous studies. This possible explanation may be that female household heads supervise their children less from initiating sexual activity than male household heads in Ethiopia. As a result, the mother's direct and indirect sources of influence on her teenage daughter's sexual conduct have been considered. Expectations that mothers who discuss sex with their daughters, mothers who keep a close watch on how their daughters act in social situations, and mothers who have developed warm, supportive relationships with their daughters are so critical.

The findings of this study highlight the importance of addressing socioeconomic and geographic disparities in early sexual initiation among adolescents in Ethiopia. The spatial variations observed suggest that contextual and structural factors play an important role in shaping adolescent sexual behaviors. These findings provide valuable evidence for policymakers and public health practitioners to design geographically sensitive interventions. Further efforts are needed to translate these findings into targeted prevention strategies that address the identified risk factors.

## 5 Strengths and Limitations of the Study

The findings of this study were not without limitations. The geographical coordinates of clusters were displaced by up to 2 km in urban areas, 5 km for most clusters in rural areas and 10 km for 1% of clusters in rural areas to protect the privacy of respondents; this could affect estimated cluster effects in the spatial analysis. To mitigate the potential impact of spatial displacement on cluster‐level estimates, this research focused on identifying broader spatial patterns and regional‐level clustering rather than precise point‐level locations. Additionally, spatial interpretations were made cautiously, emphasizing hotspot trends and spatial distributions instead of exact geographic boundaries. Therefore, conclusions drawn from this study should be interpreted in light of these considerations.

Despite the limitation, the study has the following strengths. First, it used data from a nationally representative and the largest sample of a population‐based survey, which covers all regions and administrative cities of the country. In addition, it used a robust methodology to assess the non‐stationary nature of predictor factors. The GWR results allows to explore spatially varying relationships between different predictor factors and early sexual initiation in Ethiopia. Furthermore, how the relationships between the explanatory variables and the dependent variable vary across space is visualized through maps which locate the exact locations of these factors.

## 6 Conclusion

According to this study, early sexual initiation had spatial variations across Ethiopia. The GWR analysis showed that, higher coefficients for rural residence were detected in Dire Dawa, Harari region, north eastern part of Oromia and northern part of Somali, The other important spatial predictor of early sexual initiation was poorest wealth index and its higher coefficients were detected in Addis Ababa, Benishangul Gumuz, western part of Oromia, northern part of SNNPR and north Gojjam zone of Amhara region, and being from female headed household was also a factor for early sexual initiation and higher coefficients were detected in Afar, all parts of Tigray region, the northern part of Amhara region, Benishangul Gumuz region and Wellga zone of Oromia region.

This study identified clear spatial heterogeneity in early sexual initiation among adolescents in Ethiopia, with rural residence, poor household wealth status, and female‐headed households emerging as the influential predictors. The GWR analysis revealed that the strength of these associations varied across regions, indicating that early sexual initiation is shaped by both individual‐level socioeconomic conditions and broader geographic and contextual factors. These findings highlight the importance of considering local context when interpreting adolescent sexual behavior patterns and designing interventions.

These findings underscore the need for geographically targeted adolescent health promotion and sexual and reproductive health education interventions, particularly in high‐risk areas. Strengthening community‐based and school‐based education programs that promote delayed sexual initiation, informed decision‐making, and reproductive health awareness among adolescents is essential. In addition, tailored support strategies for adolescents from economically disadvantaged and female‐headed households should be prioritized. Overall, implementing location‐specific, adolescent‐focused health promotion and education interventions may contribute to reducing early sexual initiation and improving adolescent sexual and reproductive health outcomes. By addressing early sexual initiation, these strategies have the potential to reduce early pregnancy and its associated complications and, in the long term, contribute to decreased maternal morbidity and mortality in Ethiopia.

## Author Contributions

S.D.K. designed the study, performed analysis and interpretation, and prepared the manuscript. A.A.S. made a significant contribution to the work reported, in designing the study, participated in the analysis, interpretation of the drafted manuscript, and revision the draft manuscript. All authors have read and approved the final version of the manuscript. S.D.K. had full access to all of the data in this study and takes complete responsibility for the integrity of the data and the accuracy of the data analysis.

## Funding

The authors received no specific funding for this work.

## Conflicts of Interest

The authors declare no conflicts of interest.

## Transparency Statement

The lead author Shimels Derso Kebede affirms that this manuscript is an honest, accurate, and transparent account of the study being reported; that no important aspects of the study have been omitted; and that any discrepancies from the study as planned (and, if relevant, registered) have been explained.

## Data Availability

The data used in this study are publicly available from the Demographic and Health Surveys (DHS) Program. Specifically, the 2016 Ethiopian Demographic and Health Survey (EDHS) dataset was used, which can be accessed upon reasonable request via the DHS Program website: https://www.dhsprogram.com. Researchers can register for access and request the dataset for research purposes.
